# Dielectric Elastomer Actuators with Enhanced Durability by Introducing a Reservoir Layer

**DOI:** 10.3390/polym16091277

**Published:** 2024-05-02

**Authors:** Sumin Jung, Minchae Kang, Min-Woo Han

**Affiliations:** Advanced Manufacturing & Soft Robotics Laboratory, Department of Mechanical Engineering, Dongguk University, 30 Pildong-ro 1, Jung-gu, Seoul 04620, Republic of Korea; got07242@gmail.com (S.J.); trishakang@dgu.ac.kr (M.K.)

**Keywords:** dielectric elastomer, soft actuator, soft robotics, soft materials

## Abstract

A Dielectric Elastomer Actuator (DEA) consists of electrodes with a dielectric layer between them. By controlling the design of the electrodes, voltage, and frequency, the operating range and speed of the DEA can be adjusted. These DEAs find applications in biomimetic robots, artificial muscles, and similar fields. When voltage is applied to the DEA, the dielectric layer undergoes compression and expansion due to electrostatic forces, which can lead to electrical breakdown. This phenomenon is closely related to the performance and lifespan of the DEA. To enhance stability and improve dielectric properties, a DEA Reservoir layer is introduced. Here, stability refers to the ability of the DEA to perform its functions even as the applied voltage increases. The Reservoir layer delays electrical breakdown and enhances stability due to its enhanced thickness. The proposed DEA in this paper is composed of a Reservoir layer and electrode layer. The Reservoir layer is placed between the electrode layers and is independently configured, not subjected to applied voltage like the electrode layers. The performance of the DEA was evaluated by varying the number of polymer layers in the Reservoir and electrode designs. Introducing the Reservoir layer improved the dielectric properties of the DEA and delayed electrical breakdown. Increasing the dielectric constant through the DEA Reservoir can enhance output characteristics in response to electrical signals. This approach can be utilized in various applications in wearable devices, artificial muscles, and other fields.

## 1. Introduction

There are various types of soft actuators, including pneumatic, linear, piezoelectric, Shape Memory Alloys (SMAs) and Electroactive Polymers (EAPs) [1,2]. Each of these actuators possesses unique operating principles and characteristics as well as advantages and disadvantages in terms of flexibility, force, response speed, energy conversion efficiency, and other factors [3,4,5,6,7,8].

The Dielectric Elastomer Actuator (DEA) has been proposed as one of the most utilized soft actuators among EAPs. A DEA utilizes flexible polymer materials in the middle layer with electrodes above and below. When an electric field is applied, it generates electrostatic forces, resulting in physical deformation [9,10,11]. Major applications of DEA include artificial muscles, wearable devices, smart sensors, artificial skin, etc. [12,13]. In these applications, the flexibility and deformability of DEAs play crucial roles [14].

A DEA, with its high performance, can be applied as an actuator. Artificial muscles created using DEAs can mimic the movements and flexibility of natural muscles [15,16]. Through this, robots capable of interacting safely and smoothly with humans and performing complex tasks at a level similar to biological organisms can be developed [17]. DEA-based artificial muscles resemble human muscles in terms of strength, strain, and actuation pressure, and these characteristics make them applicable in a wide range of fields [18]. These DEAs can detect and assist human movements simultaneously [15,16]. Under electrical stimulation, DEAs exhibit significant deformation and rapid response times [19,20]. Leveraging these characteristics, they can be utilized in creating comfortable wearable devices [10,21]. The application of DEAs with delayed breakdown could lead to the development of wearable devices that enhance wearer strength and endurance, with these elements being capable of being sustained for long periods. The high dielectric constant of DEAs enables more sensitive and accurate detection capabilities that can be utilized in sensor development, which requires the detection of minute changes.

Various research efforts have been conducted to enhance the performance of DEAs, aiming to utilize them in multiple domains [22]. Structural improvements of the actuator directly impact the enhancement of DEA performance. Research on enhancing electrical and mechanical performance through multilayered structures has been ongoing [23,24,25]. Multilayered DEAs increase the dielectric layers to enhance electrical force and achieve greater deformation. The performance of the dielectric layer can also be improved depending on the material used between the electrodes of the DEA [26]. Various materials for dielectrics, such as high dielectric constant, low viscosity, and high thermal stability, are being studied [27,28,29]. The utilization of these new dielectric materials contributes to increasing the dielectric constant of DEA, reducing electrical resistance, expanding the operating temperature range, and enhancing resistance to electrical breakdown. Efficient control algorithms are utilized for precise control of DEAs. These algorithms predict the dynamic behavior of DEAs and adjust the voltage to achieve optimal performance. Control algorithms enable fast response times and self-sensing, as well as precise position control of DEAs and enhanced electrical stability [30].

In this paper, the focus is on structural improvements to achieve enhancing durability and improving the dielectric properties of DEAs. We aim to enhance the dielectric properties of DEAs by applying a multilayered structure. Additionally, we introduce a new intermediate layer called the ‘Reservoir’ layer, which is composed of carbon grease and independently configured without electrode connections. By utilizing independently configured conductive regions, we increase the dielectric properties of DEAs while delaying electrical breakdown. We observe deformation characteristics by varying the size ratio or structure of the ‘Reservoir’ layer compared to the electrode layer. Such structural innovations can be applied in various fields, such as artificial muscles, wearable devices, smart sensors, and artificial skin, through utilizing DEAs.

## 2. Materials and Methods

In this study, for the fabrication of a Reservoir DEA, we utilize flexible electrodes such as carbon grease, copper tape for electrode connections, VHB film, and a guide ring printed with a 3D printer for pre-stretching the VHB. In this study, carbon conductive grease 846 (MG Chemicals, Burlington, ON, Canada) is used, and its resistivity is 63 Ω·cm. Carbon grease serves as the material for flexible electrodes. It reduces the contact resistance between the electrode and the dielectric layer and increases the flexibility of the electrode, enabling a more uniform distribution of the electric field. By increasing the adhesion of the electrodes, carbon grease can reduce energy loss in the dielectric layer and overall enhance the performance of DEA.

Copper tape, known for its excellent conductivity, is used as the electrode material to achieve low resistance when used in the dielectric layer. Its flexibility and strong conductivity allow it to be easily connected to flexible electrodes.

In this study, VHB 4910 (3M, Maplewood, MN, USA) with a width of 80 mm and a thickness of 1 mm was selected. VHB film possesses characteristics such as a very high elastic energy density (3.4 J/g), large actuation strain as high as 380% in area, and the ability to withstand stress of up to 7.2 MPa [31]. VHB film is a high-performance adhesive film known for its outstanding adhesion and high flexibility. Additionally, VHB film has the advantage of allowing for the fabrication of DEAs through a simple process of prestretching and stacking, without the need for other complex procedures like spin coating processes. Therefore, VHB film was chosen for its ease and convenience in creating the various DEAs that will be discussed in the paper.

These materials and components are crucial for the fabrication of a Reservoir DEA, contributing to its overall performance and functionality in various applications [32]. DEA fabrication with VHB involves a prestretching process, allowing VHB, an acrylic-based elastomer, to achieve a maximum strain of up to 215%, further enhancing its performance [19]. In this study, the VHB film was manually prestretched. An approximately 200% prestretch was performed centered around a radially 3D-printed guide ring. Additionally, the excellent adhesion of VHB film enables easy stacking of layers with flexible electrodes, contributing to increasing the overall dielectric properties of the DEA structure. To facilitate the prestretching process of VHB film, a ring-shaped structure was created that was made of PLA material and printed using an FDM 3D printer.

The basic guide ring was manufactured with an outer diameter of 63 mm, an inner diameter of 58 mm, and a thickness of 1 mm. The bigger guide ring was made with an outer diameter of 76 mm, an inner diameter of 71 mm, and a thickness of 1 mm. Therefore, the VHB films for the outer layers of the three-layered DEA, excluding the middle layer, were prestretched to a diameter of 63 mm before being adhered. The thickness of the VHB film after prestretching was approximately 0.2 mm.

To create a 1-layer DEA, first the VHB film is prestretched and attached to a guide ring. Then, a film cut into the desired shape using laser cutting is attached to the prestretched VHB film to apply carbon grease only to specific areas. Carbon grease is then applied, and copper tape is attached to the top and bottom as electrode layers.

For a 2-layered DEA, two structures with VHB film prestretched on a guide ring are made. Carbon grease is applied bidirectionally to one of these structures. One is then connected to copper tape to become an electrode layer, and the other becomes a Reservoir layer. The other VHB film structure is then adhered to the one with applied carbon grease, taking care to avoid air bubbles. After this, carbon grease is applied to the adhered VHB film and copper tape is attached to the top and bottom to complete it.

To make a 3-layered DEA, two structures with VHB film prestretched on a guide ring are created, and one more is then made on a bigger guide ring. Carbon grease is applied to the top and bottom of the VHB film structure on the bigger guide ring in the desired shape. Both layers of carbon grease will become Reservoir layers. The remaining two VHB film structures are then adhered to the one with applied carbon grease, carefully avoiding air bubbles. Carbon grease is then applied to each of the two adhered VHB films, and copper tape is attached to the topmost and bottom parts to finish the assembly.

**Figure 1:**
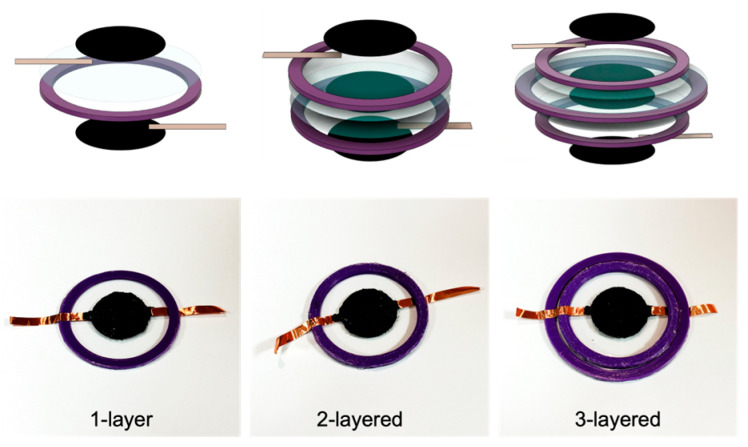
Conceptual diagrams and appearances of the three types of specimens: 1-layer, 2-layered, and 3-layered.

**Figure 2:**
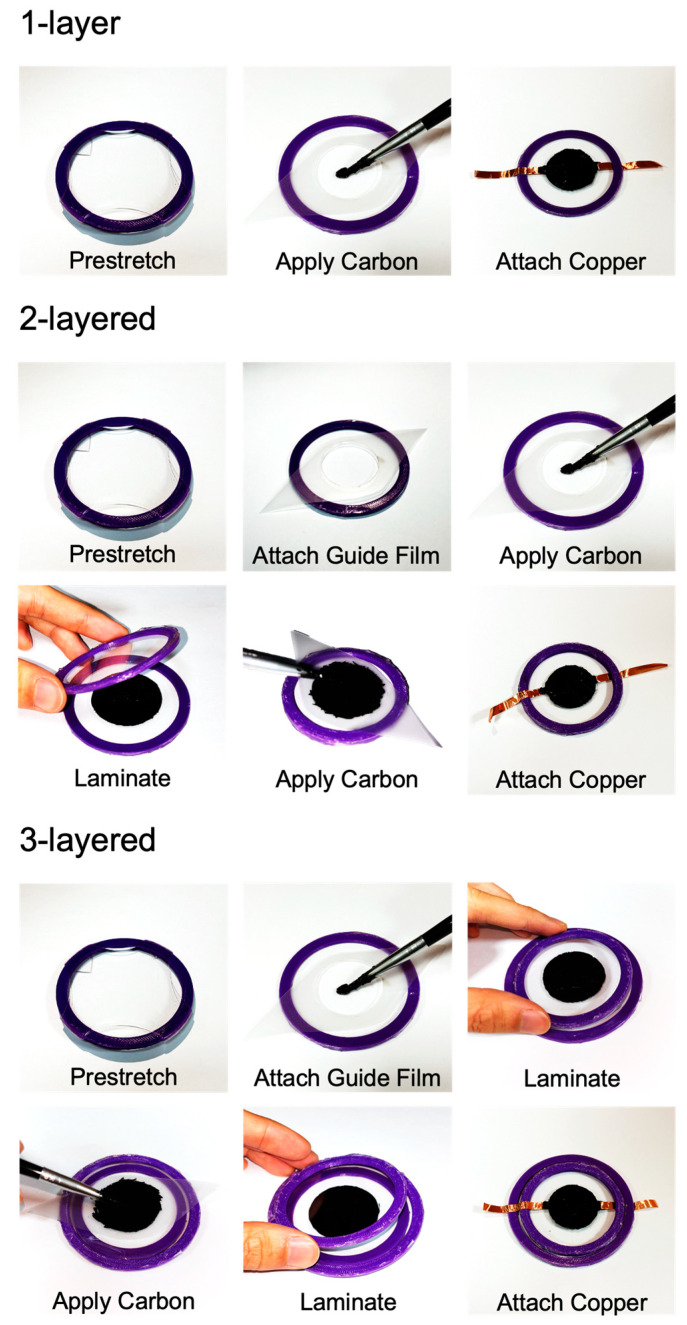
The fabrication process of the three types of DEAs: 1-layer, 2-layered, and 3-layered.

Based on the number of layers of VHB stacked, three types of samples were fabricated: 1-layer, 2-layered, and 3-layered (see Figure 1). In the case of 1-layer DEA fabrication, VHB is placed in the middle, with carbon grease electrodes being applied on both sides. Reservoir is not applied in this case. Next, for a 2-layered DEA, prestretched VHB is placed on both sides with carbon at both ends and a reservoir in the middle. Lastly, for a 3-layered DEA, prestretched VHB is placed on both sides with carbon at both ends and a reservoir in the middle. For this purpose, a larger guide ring is used (see Figure 2).

**Figure 3:**
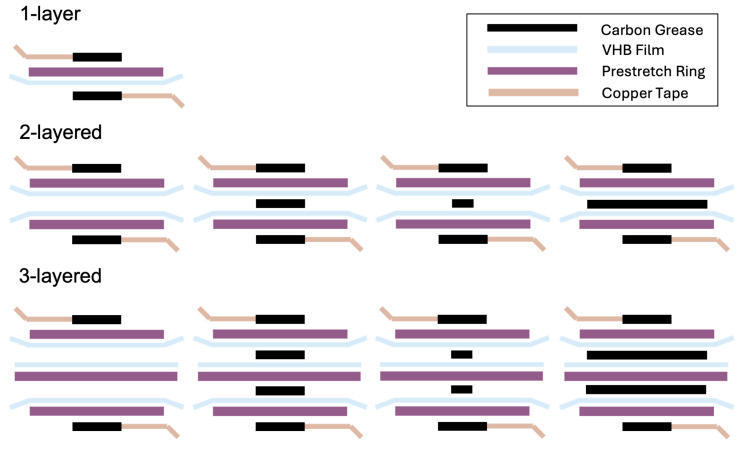
The fabrication methods of the Reservoir layer: 1-Layer, 2-Layered, and 3-Layered.

Additionally, the following four cases were fabricated based on the presence and size of the Reservoir layer: when there is no Reservoir layer, when the Reservoir layer is smaller, equal in size, or larger than the electrode layer (see Figure 3). Furthermore, to observe variations based on shape, electrode layers and Reservoir layers of the same size were fabricated in the shapes of circles, triangles, and squares.

During the experiment, the dielectric elastomer is connected to a high-voltage power supply. In this setup, the dielectric elastomer is connected to copper tape only on the top and bottom layers of the carbon grease, and no copper tape is connected to the carbon grease in the Reservoir layer. The voltage is increased from 0 kV to 10 kV in increments of 0.5 kV using the high-voltage power supply, and changes in diameter are then measured.

## 3. Results

### 3.1. One-Layer DEA

**Figure 4:**
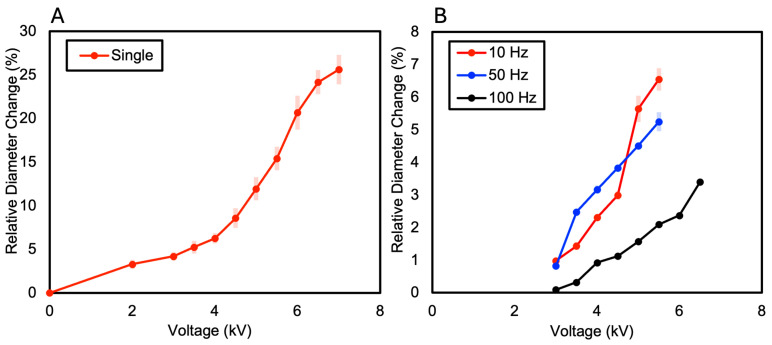
(**A**) The relative diameter changes in the 1-layer DEA as the voltage increases; (**B**) The relative diameter changes in the 1-layer DEA at a frequency of 10 Hz, 50 Hz, and 100 Hz as the voltage increases.

**Figure 5:**
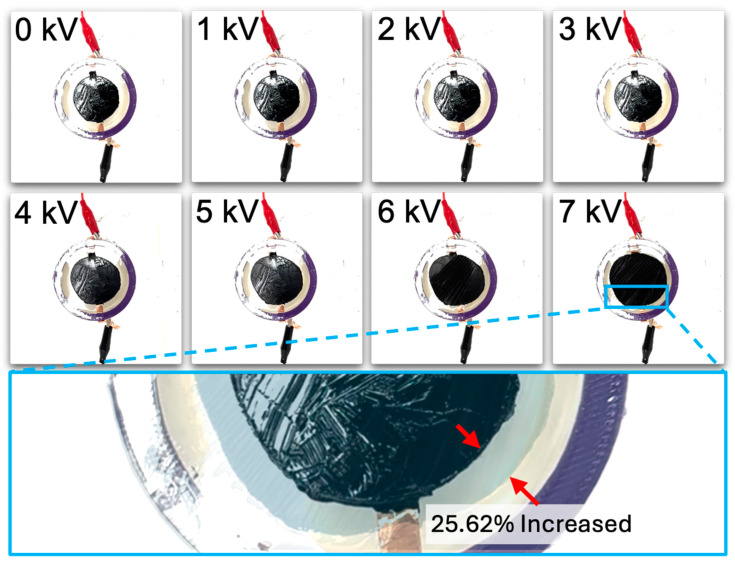
The changes in the one-Layer DEA as the voltage increases. The images at 0 kV and at the maximum diameter change were overlaid with adjusted transparency. The red arrows were added to make it easy to identify the difference between the original diameter and the maximally changed diameter.

In this paper, the relative diameter change ($\;\frac{{∆D}_{avg}}{D_{o}})$ was calculated and compared to measure variations in each specimen. This is a value represented as a ratio, calculated by averaging the changes in diameter (${∆D}_{avg})$ relative to the original diameter of the specimen ($D_{o})$ and then dividing by the original diameter. A dielectric elastomer consisting of a single layer of VHB film exhibited a relative diameter change of 25.62% at 7 kV, after which electrical breakdown occurred at 7.5 kV, as shown in Figure 4A and Figure 5. When measuring the relative diameter change according to frequency variations of 10 Hz, 50 Hz, and 100 Hz, the results were as follows: at 10 Hz, a relative diameter change of 6.54% was observed at 5.5 kV; at 50 Hz, a relative diameter change of 5.24% was observed at 6 kV; and at 100 Hz, a relative diameter change of 3.4% was observed at 6.5 kV (see Figure 4B). Electrical breakdown occurred at 6 kV for both 10 Hz and 50 Hz frequencies and at 7 kV for 100 Hz frequency.

### 3.2. Two-Layered DEA

**Figure 6:**
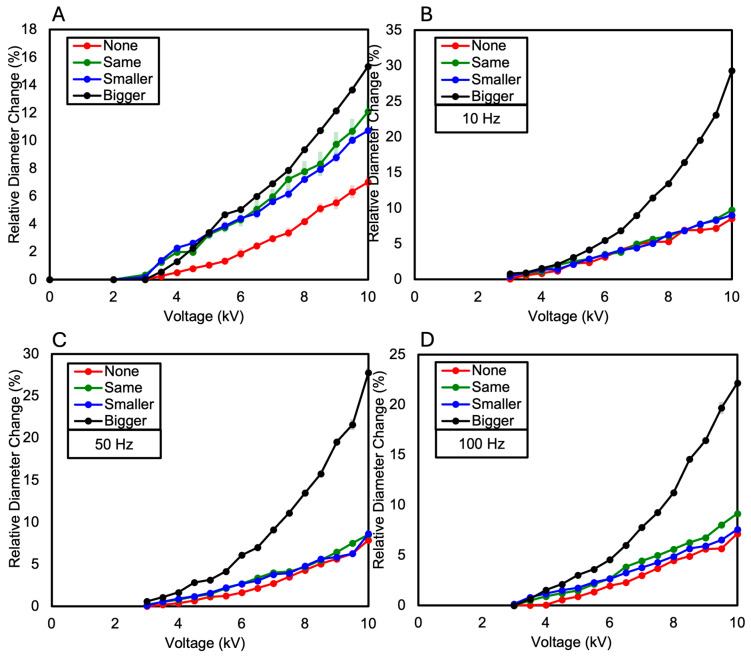
(**A**) The relative diameter change in the two-Layered DEA as the voltage increases; (**B**) the relative diameter change in the two-Layered DEA at a frequency of 10 Hz as the voltage increases; (**C**) the relative diameter change in the two-Layered DEA at a frequency of 50 Hz as the voltage increases; (**D**) the relative diameter change in the two-Layered DEA at a frequency of 100 Hz as the voltage increases.

**Figure 7:**
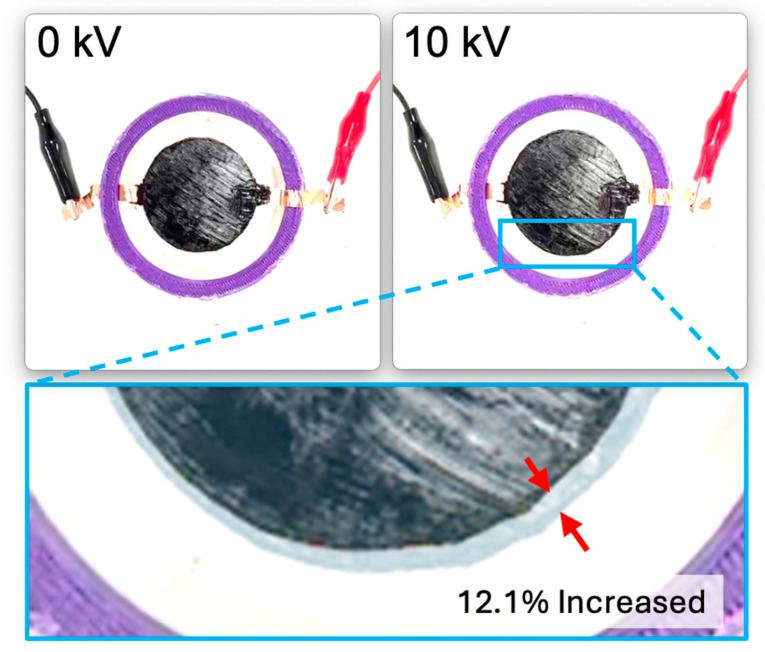
The changes in the two-Layered DEA as the voltage increases. The image of blue box is the images at 0 kV and at the maximum diameter change were overlaid with adjusted transparency. The red arrows were added to make it easy to identify the difference between the original diameter and the maximally changed diameter.

**Figure 8:**
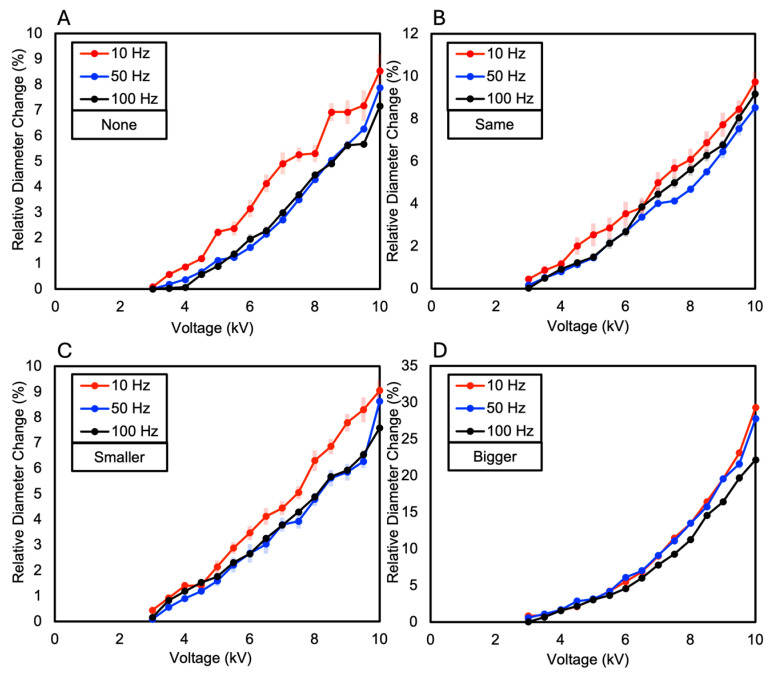
The relative diameter change as the voltage increases (**A**) when the two-Layered DEA does not have a Reservoir layer; (**B**) when the diameter of the Reservoir layer in the two-Layered DEA is the same as that of the electrode layer; (**C**) when the diameter of the Reservoir layer in the two-Layered DEA is smaller than that of the electrode layer; (**D**) when the diameter of the Reservoir layer in the two-Layered DEA is larger than that of the electrode layer.

The dielectric elastomer composed of two layers of VHB film was divided into the following four samples based on the presence and size of the Reservoir layer: when there is no Reservoir layer, when the Reservoir layer is the same size as the electrode layer, when the Reservoir layer is smaller than the electrode layer, and when the Reservoir layer is larger than the electrode layer. As a result, the relative diameter change was observed in the order of increasing size ratio of the Reservoir layer compared to the electrode layer. When the diameter of the Reservoir layer was larger than the electrode layer, a relative diameter change of 15.35% was observed. When the Reservoir layer was the same size as the electrode layer, a relative diameter changes of 12.1% was observed. When the Reservoir layer was smaller than the electrode layer, a relative diameter changes of 10.74% was observed. When there was no Reservoir layer, a relative diameter changes of 7.02% was observed (see Figure 6A and Figure 7). These results were consistent with the measurements of relative diameter changes for the four types of samples according to frequency variations of 10 Hz, 50 Hz, and 100 Hz, showing that the relative diameter change increased in the order of increasing size ratio of the Reservoir layer compared to the electrode layer, as shown in Figure 6B–D. Observing the relative diameter change with the increase in voltage for each frequency (10 Hz, 50 Hz, 100 Hz) per sample, although there were no significant differences among all frequencies, it was generally observed that the relative diameter change increased in the order of 10 Hz, 50 Hz, and 100 Hz (see Figure 8).

### 3.3. Three-Layered DEA

**Figure 9:**
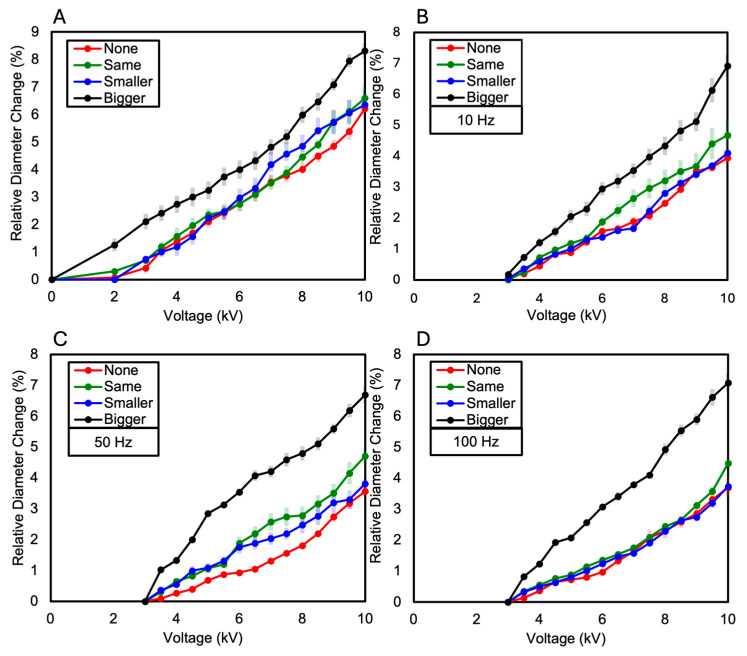
(**A**) The relative diameter change in the three-Layered DEA as the voltage increases; (**B**) the relative diameter change in the three-Layered DEA at a frequency of 10 Hz as the voltage increases; (**C**) the relative diameter change in the three-Layered DEA at a frequency of 50 Hz as the voltage increases; (**D**) the relative diameter change in the three-Layered DEA at a frequency of 100 Hz as the voltage increases.

**Figure 10:**
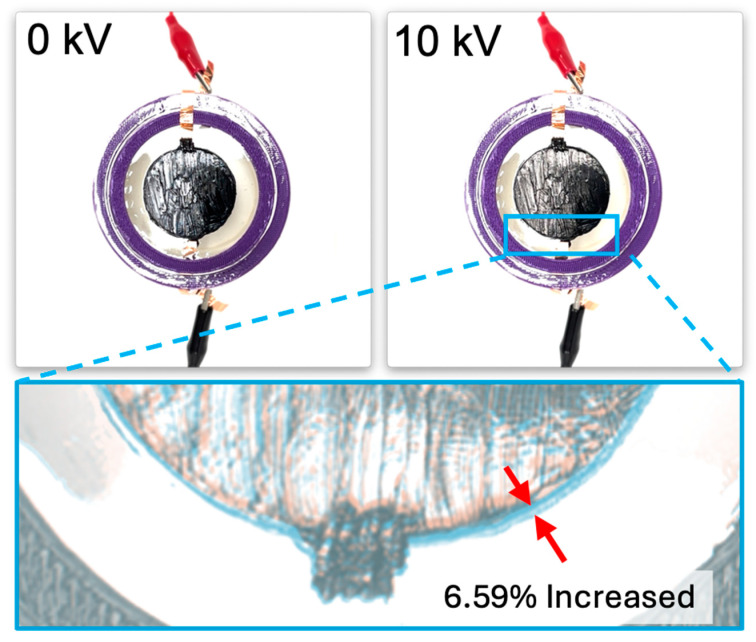
The changes in the three-Layered DEA as the voltage increases. The image of blue box is the images at 0 kV and at the maximum diameter change were overlaid with adjusted transparency. The red arrows were added to make it easy to identify the difference between the original diameter and the maximally changed diameter.

**Figure 11:**
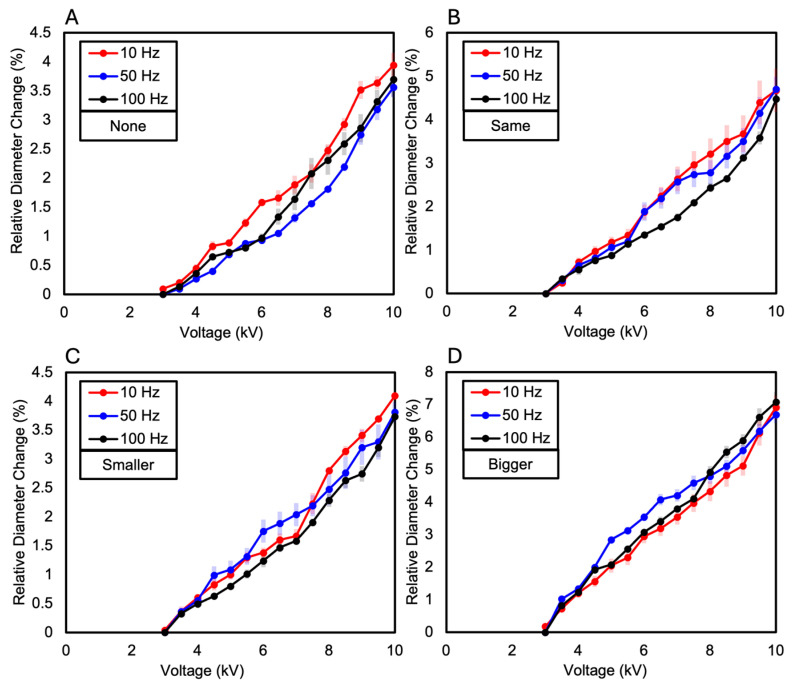
The relative diameter change as the voltage increases (**A**) when the three-Layered DEA does not have a Reservoir layer; (**B**) when the diameter of the Reservoir layer in the three-Layered DEA is the same as that of the electrode layer; (**C**) when the diameter of the Reservoir layer in the three-Layered DEA is smaller than that of the electrode layer; (**D**) when the diameter of the Reservoir layer in the three-Layered DEA is larger than that of the electrode layer.

The dielectric elastomer composed of three layers of VHB film was divided into the following four samples, like the two-layered configuration: when there is no Reservoir layer, when the Reservoir layer is the same size as the electrode layer, when the Reservoir layer is smaller than the electrode layer, and when the Reservoir layer is larger than the electrode layer. In each sample, the Reservoir layer consisted of two layers, and their sizes were made equal. As a result, similar to the two-layered configuration, the relative diameter change was observed in the order of increasing size ratio of the Reservoir layer compared to the electrode layer. When the diameter of the Reservoir layer was larger than the electrode layer, a relative diameter change of 8.31% was observed. When the Reservoir layer was the same size as the electrode layer, a relative diameter change of 6.59% was observed. When the Reservoir layer was smaller than the electrode layer, a relative diameter change of 6.35% was observed. When there was no Reservoir layer, a relative diameter change of 6.22% was observed (see Figure 9A and Figure 10). When measuring the relative diameter change for the four types of samples according to frequency variations of 10 Hz, 50 Hz, and 100 Hz, the relative diameter change values were quite similar for the “none”, “same”, and “smaller” Reservoir layer samples. However, for the “bigger” Reservoir layer sample, a superior relative diameter change was observed compared to the other samples at all three frequencies. However, it decreased by approximately 45% compared to the relative diameter change of the two-layered configuration, as shown in Figure 9B–D. Observing the relative diameter change with the increase in voltage for each frequency (10 Hz, 50 Hz, and 100 Hz) per sample, although there were no significant differences among all frequencies, it was observed that the relative diameter change increased in the order of 10 Hz, 50 Hz, and 100 Hz for the samples without a Reservoir layer and for the samples where the Reservoir layer’s diameter was the same as or smaller than the electrode layer (see Figure 11).

### 3.4. Normalization of DEAs

**Figure 12:**
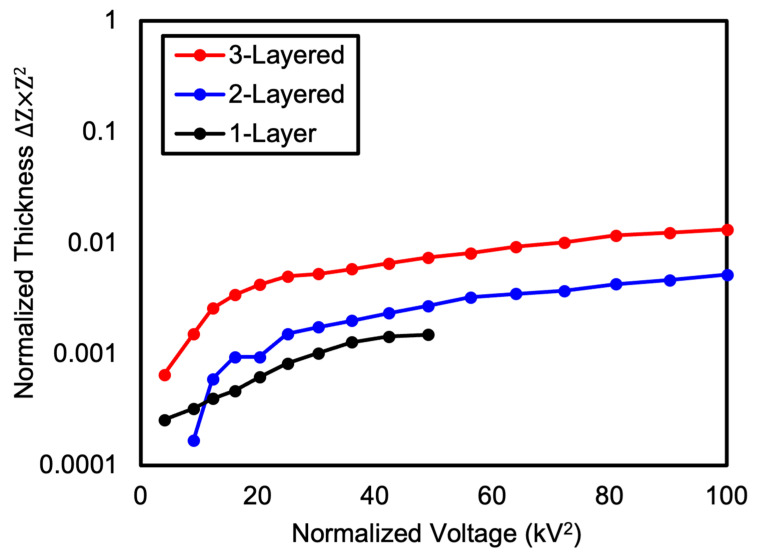
Change in normalized thickness of one-layer, two-layered and three-layered DEAs with increasing voltage.

To further investigate the effect of DEA thickness and the introduction of Reservoir layers on the voltage-induced deformation behavior, we normalized the data by calculating the normalized thickness as the product of the voltage-dependent thickness change and the square of the initial thickness for each DEA layer. This normalized thickness was then plotted against the square of the applied voltage (Figure 12). The graph shows that the three-layered DEA exhibited the largest change in normalized thickness, followed by the two-layered and one-layer DEAs, respectively. The three-layered DEA with two reservoir layers showed the greatest change in normalized thickness, while the two-layered DEA with one Reservoir layer and the one-layer DEA without a Reservoir layer exhibited relatively smaller changes. This indicates that the dielectric properties of DEAs improve as the number of Reservoir layers increases. As the data points of all DEAs show a similar trend with respect to the square of the applied voltage in the normalized graph, it can be concluded that the deformation behavior of DEAs is influenced by the square of the applied voltage.

### 3.5. Experiment Summary

The one-layer dielectric elastomer exhibited a relative diameter change of 25.62% at 7 kV, followed by an electrical breakdown at 7.5 kV. The relative diameter changes due to frequency variations were 6.54% at 10 Hz, 5.24% at 50 Hz, and 3.4% at 100 Hz. Electrical breakdown occurred at 6 kV for 10 Hz and 50 Hz frequencies, and then at 7 kV for the 100 Hz frequency.

For the two-layered dielectric elastomer, the relative diameter change varied based on the size of the Reservoir layer. The larger the diameter of the Reservoir layer compared to the electrode layer, the higher the relative diameter change. Specifically, when the Reservoir layer was larger than the electrode layer, the relative diameter change was 15.35%; when they were the same size, it was 12.1%; when the Reservoir layer was smaller, it was 10.74%; and when there was no Reservoir layer, it was 7.02%. Consistently, in the experimental results according to frequency variations of 10 Hz, 50 Hz, and 100 Hz, larger Reservoir layer size ratios resulted in greater relative diameter changes. The presence of the Reservoir layer contributed to delaying electrical breakdown and enhancing dielectric properties.

Similarly, the three-layered dielectric elastomer exhibited results akin to the two-layered configuration. The relative diameter change varied based on the size of the Reservoir layer, with larger Reservoir layer size ratios resulting in higher relative diameter changes. In the frequency variation experiments, larger Reservoir layer size ratios consistently led to greater relative diameter changes. However, compared to the two-layered configuration, the three-layered configuration showed a decrease in relative diameter change. This decrease is attributed to the increased stiffness due to the thicker VHB layer in the three-layered configuration. Nonetheless, the increased stiffness in the three-layered configuration suggests that it may delay electrical breakdown further, while increasing the size of the Reservoir layer could complement dielectric properties.

Furthermore, experiments were conducted with two-layered dielectric elastomers of identical size for the Reservoir and electrode layers in triangular, square, and circular shapes. The circular shape exhibited an area change rate of 34.54% at 10 kV, the square shape exhibited an area change rate of 14.54% at 10 kV, and the triangular shape exhibited an area change rate of 7.15% at 10 kV.

## 4. Conclusions

This paper proposes a multilayer DEA with an introduced Reservoir layer and examines its behavioral characteristics. It was confirmed that the proposed reservoir DEA contributes to delaying electrical breakdown compared to conventional DEAs and enhances dielectric properties. Notably, the introduction of the Reservoir layer significantly enhances the durability of the polymer by increasing its thickness, which prevents frequent electrical breakdowns. Although this configuration results in a slight decrease in the dielectric constant compared to conventional DEAs, it substantially improves the dielectric properties, leading to enhanced durability. This balance between maintaining dielectric properties and improving durability represents a significant advancement over existing DEA technologies. To verify its performance characteristics, experiments were conducted to compare and evaluate the performance of one-layer, two-layered, and three-layered structured DEAs, revealing that the presence and size of the Reservoir layer positively impact DEA performance. DEAs play a significant role in various fields such as artificial muscles, wearable devices, smart sensors, and artificial skin. This research suggests a new approach to overcome the existing limitations of DEAs and significantly enhance their performance, thereby expanding the possibilities for technological advancement and applications. By improving both the electrical stability and deformation rates in particular, more effective use in a wider range of applications is anticipated. When a voltage is applied to a circular DEA, it expands while maintaining its shape and then returns to its original state. However, there were limitations in uniformly applying the carbon grease, resulting in deviations from the perfect original shape upon expansion. Future work should consider methods to uniformly apply carbon grease. Based on the outcomes of this research, future work could explore various methods to enhance DEA performance by diversifying the design of the Reservoir layer using different materials and structures. Additionally, focusing on practical tests in various application fields could validate the practicality of this new DEA and expand its application scope. Furthermore, expanding research directions towards sustainability and environmental impact considerations could contribute to the development of environmentally friendly yet high-performance soft actuators.

## Data Availability

Data are contained within the article.
